# Implementing measurement based care in community mental health: a description of tailored and standardized methods

**DOI:** 10.1186/s13104-018-3193-0

**Published:** 2018-01-27

**Authors:** Cara C. Lewis, Ajeng Puspitasari, Meredith R. Boyd, Kelli Scott, Brigid R. Marriott, Mira Hoffman, Elena Navarro, Hannah Kassab

**Affiliations:** 1 Kaiser Permanente Washington Health Research Institute, 1730 Minor Ave, Suite 1600, Seattle, WA 98101 USA; 2 Department of Psychological and Brain Sciences, Indiana University, 1101 East 10th St, Bloomington, IN 47401 USA; 3 Department of Psychiatry and Behavioral Sciences, Harborview Medical Center, University of Washington, 325 9th Ave, Box 354946, Seattle, WA 98104 USA; 4 Department of Psychiatry and Psychology, Mayo Clinic, 200 First St SW, Rochester, MN 55905 USA; 5 Department of Psychological Sciences, University of Missouri, 210 McAlester Hall, Columbia, MO 65211 USA; 6 Centerstone Research Institute, 409 West 1st St, Bloomington, IN 47403 USA

**Keywords:** Implementation, Tailored, Standardized, Measurement based care, Depression, Community mental health

## Abstract

**Objective:**

Although tailored implementation methods are touted as superior to standardized, few researchers have directly compared the two and little guidance regarding the specific details of each method exist. Our study compares these methods in a dynamic cluster randomized trial seeking to optimize implementation of measurement based care (MBC) for depression in community behavioral health. This specific manuscript provides a detailed, replicable account of the components of each multi-faceted implementation method.

**Results:**

The standardized best practice method includes training, consultation, a clinical guideline, and electronic health record enhancements with the goal to optimize the delivery of MBC with fidelity. Conversely, the tailored, customized and collaborative method is informed by recent implementation science advancements and begins with a needs assessment, followed by tailored training that feeds back barriers data to clinicians, the formation of an implementation team, a clinician-driven clinic-specific guideline, and the use of fidelity data to inform implementation team activities; the goal of the tailored condition is to ensure the intervention and implementation strategies address unique factors of the context. The description of these methods will inform others seeking to implement MBC, as well as those planning to use standardized or tailored implementation methods for interventions beyond behavioral health.

**Electronic supplementary material:**

The online version of this article (10.1186/s13104-018-3193-0) contains supplementary material, which is available to authorized users.

## Introduction

Measurement based care is an evidence-based practice (EBP) that involves the routine use of standardized assessment results (i.e., Patient Health Questionnaire, PHQ-9 for depression severity [1]) to guide psychotherapy practice [2], but this practice is used by fewer than 20% of behavioral health clinicians in the United States [3–5]. Implementation science has emerged to address this research-to-practice gap. There is mounting evidence that discrete implementation strategies are insufficient, but it is unclear how to best package multifaceted strategies. A Cochrane review indicated variable impact of tailored implementation methods over standardized approaches in healthcare settings [6]. Optimal methods to identify determinants and inform tailoring remain unknown [7]. This manuscript provides a detailed and replicable description of the methods used in an ongoing study that is comparing standardized versus tailored implementation of measurement based care (MBC) on clinician-level (fidelity) and client-level (depression severity) outcomes in community behavioral health settings. See Table 1 for an overview of methods.

**Table 1:** Comparison of conditions

Contextual factor	Implementation strategies	Standardized, “best practices”	Tailored, “customized and collaborative”
Resources	Needs assessment Electronic Health Record (EHR) Enhancements	Client completion of paper PHQ-9 and score entered in EHR for review by the clinician	Client Completion of paper PHQ-9 and score entered in EHR for review by the clinician
Networks & Linkages	Teams were formed and met triweekly	All clinicians were invited to attend	Opinion leaders and champions were invited to attend
Policies and Incentives	Guideline for PHQ-9 administration frequency	Each session with client	Determined by implementation teams, specific to each site
Norms & Attitudes	Initial MBC training Audit & Feedback with fidelity data	Standardized training material Penetration data to monitor fidelity, but not provided to clinicians	Tailored training material targeting identified barriers from the needs assessment Penetration data to inform tailored implementation
Structure & Process	Progress note modifications in EHR	Graph available for score review	Graph available for score review
Media & Change Agents	Triweekly meetings with external experts	Consultation focused on promoting MBC fidelity: (1) session-by-session administration of PHQ-9; (2) clinician score to inform session; (3) discussion of scores with clients in session. Clinicians were offered tips on targeting lack of progress	Consultation focused on targeting contextual barriers, with emphasis placed on fidelity to site-specific guideline

The implementation strategies were selected to map onto the six domains of the context of diffusion as outlined in the *Framework for Dissemination* [10]

## Main text

### Setting and participants

The study protocol is published elsewhere [8]. The main study is a dynamic cluster randomized trial (12 sites randomized to one of four cohorts and then randomized to condition: standardized or tailored) in partnership with the nation’s largest community-based outpatient behavioral health service provider. Participants consist of both clinicians and clients. Clinicians are representative of Masters level counselors (primarily female, Caucasian, less than 50% licensed). Clients are adults with a diagnosis of depression (primary or secondary) and a PHQ-9 score greater than 10, receiving psychotherapy from a participating clinician.

### Standardized implementation method

The standardized method was conceptualized as a “best practices” approach to implementation, incorporating six discrete implementation strategies [9]: (1) electronic health record (EHR) enhancements; (2) a needs assessment; (3) training; (4) MBC guidelines; (5) forming a team; and (6) tri-weekly group consultation. The strategies in this condition were led by a clinical psychologist with MBC expertise and took place over a 5-month active implementation period, followed by a 10-month sustainment period.

#### EHR enhancements

The PHQ-9 questions were embedded in the EHR to allow clinicians to transfer clients’ scores from paper form to their individual charts. The EHR calculated total PHQ-9 scores and produced symptom trajectories over time. Clinicians used this graph to monitor outcomes and communicate symptom change (or lack thereof) with clients or other providers. Several self-report questions were also embedded into the progress note to collect MBC fidelity data, such as whether clinicians discussed scores with clients, or indicate why the PHQ-9 was not completed. Clinicians were encouraged but not required to answer these questions.

#### Needs assessment

All clinicians completed a battery of self-report measures and a subset attended a focus group during a baseline needs assessment to reveal contextual factors of influence across six domains (i.e., norms & attitudes; structure & process; resources; policies & incentives; networks & linkages; media & change agents) guided by the Framework for Dissemination [10]. Focus group members were identified by clinic administrators using purposeful sampling to achieve extreme variation [11]. The results from the needs assessment were used to characterize clinics but not inform the implementation process, except that several measures were used to prioritize clinicians for attending the consultation meetings (see below).

#### Initial training

Clinicians attended a 4-h training 1 month after the needs assessment. The primary goal was to introduce clinicians to MBC and the PHQ-9 and build foundational knowledge and skill regarding the three core components: (1) administer the PHQ-9, (2) review score graphs, and (3) discuss scores with the client. The training covered: MBC introduction and scientific support; core components of MBC; PHQ-9 scientific support; and clinical tips for addressing lack of progress. Active learning strategies were included in the training guided by adult learning principles [12, 13]. The trainer provided didactic content, modeled how to administer the PHQ-9, engaged clinicians in practicing MBC core components and offered immediate feedback, and showed videos on how to use MBC with challenging clients. Clinicians were invited to ask questions and participate in group discussion. Clinicians earned four CEUs and the clinic received equivalent financial incentives to cover productivity for time spent in training.

#### Guideline

The recommended guideline to implement MBC was discussed during the initial training and throughout the consultation meetings. This guideline was informed by the available literature (e.g., [14]) stating that clinicians ought to administer, review, and discuss the PHQ-9 with depressed adult clients in the beginning of every session. This guideline was presented to clinicians as a strong recommendation not a requirement.

#### Building consultation teams

Although all clinicians were invited to join the consultation meetings, opinion leaders (i.e., individuals who hold influence in a social network) and champions (i.e., individuals who vocally support an innovation), as determined by self-report measures at baseline (i.e., Sociometric Survey [15] and Opinion Leadership Scale [16]), were invited to attend the consultation meetings and their schedules were prioritized, noting that they play a pivotal role in their clinic.

#### Consultation team meetings

It is well-documented that training alone is insufficient for changing behavior and consultation is needed to increase skill and support implementation [17]. The primary goal of the consultation meetings was to facilitate clinician use of MBC with fidelity to the core components. Each meeting was 60 min and was led by the external consultant via video conference. The consultant was proscribed from focusing on problem solving contextual implementation barriers. On average, six consultation meetings were held in each participating clinic during the 5-month active implementation period. For each meeting, two clinicians presented examples of clients with whom they tried but struggled to implement MBC. A standardized case consultation form was completed prior to the meeting to provide the consultant with context and specific questions (see Additional file 1). Active learning strategies (e.g., group discussion, modeling, practice + feedback) were used to engage clinicians. Clinicians received 1 CEU for each meeting.

### Tailored implementation method

The tailored implementation method was conceptualized as “customized and collaborative” and it was informed by the Dynamic Sustainability Framework [18], as well as the Dynamic Adaptation Process [19], both of which acknowledge that evidence based practices may require adaptation to best fit the context and implementation strategies selected by a diverse group of stakeholders to target emerging barriers may optimize sustainment. The tailored method included the same six implementation strategies as the standardized method to control for dose/time and resources. However, tailoring occurred in the initial training, in the MBC guideline development, and by the implementation teams.

#### Needs assessment

Similar to the standardized method, all clinicians completed a battery of self-report measures, and a subset of clinicians participated in focus groups. However, director interviews and clinic tours were also conducted by the research team as part of the rapid ethnography [20] to understand the unique clinic contexts. Needs assessment results were used to tailor the initial training and the data was fed back to clinicians to reveal barriers (e.g., insufficient administrative support, lack of integration with current workflow) and facilitators (e.g., leadership support, prior experience with MBC) specific to their site.

#### Initial training

Tailored MBC training was modeled after the same 4-h, expert-led, didactic workshop format as the standardized condition, but training in the tailored condition was customized to address clinic-specific barriers identified in the needs assessment. Training was tailored either by adjusting content to be more relevant to the specific clinic, or altering the structure of the training to include other discussions/examples/activities. For instance, at clinics where clinicians noted that they did not view evidence-based practices as clinically useful or as important as clinical judgment, research evidence was presented to demonstrate that clinicians often overestimate client progress (tailoring of content), or audio recordings of clinicians discussing the clinical utility of the PHQ-9 were incorporated into training (tailoring of structure). Clinic-specific barriers and facilitators were presented at the end of training in the tailored, but not the standardized, condition. The trainings in each condition were conducted by different psychologists to avoid contamination.

#### Forming implementation teams

In order to maximize diversity in team composition and their ultimate impact, implementation teams were formed using a combination of approaches (i.e., network analysis using sociometric survey, attitudinal measures, self-nominations) to identify key individuals to drive and support the MBC implementation. Implementation teams consisted of five to eight members typically including clinic director(s), clinicians, and the study PI (who served as facilitator). Team composition reflected membership from different tiers (e.g., director, team leader, clinician, and office professional) and different teams within each clinic. Social network analysis was used to identify two opinion leaders from each clinic’s baseline advice networks; specifically, opinion leaders who had the highest in-degree centrality (a measure of influence; [21]) and spanned the network. Two attitudinal measures [5] were used to identify two clinicians who strongly endorsed positive attitudes toward MBC to serve as champions on the team. Teams were charged with advancing MBC through the phases of implementation, communicating with other stakeholders, engaging in data-based decision making, increasing buy-in, problem-solving, and identifying barriers and finding solutions, for instance [22].

#### Tailored guideline

Implementation teams were given the choice to tailor the MBC guideline at their clinic rather than follow the standardized recommendation. Some examples of guideline tailoring include the expansion of PHQ-9 administration to clients as young as twelve and to clients without depression. One clinic deemed the first week of each month “assessment week” and focused on giving the PHQ-9 to every client during that week only. Another clinic gave each clinician a list of ten clients to focus PHQ-9 administration, with the intention of allowing clinicians to become comfortable with the PHQ-9 before expanding use.

#### Implementation team meetings

The primary task of implementation teams was to choose strategies to support MBC use during their tri-weekly meetings and enact them over the course of 5 months. While the specific activities of each team differed based on the barriers identified and strategies chosen, there were three common components across teams. First, each team assigned positions of chair, secretary, and evaluation specialist. The chair generated an agenda and led meetings; the secretary took notes and distributed to other members of the team; and the evaluation specialist received, reviewed and presented penetration data (discussed below). Several teams also opted to create a communication specialist position who was responsible for communicating decisions made by the team with others at the clinic.

Second, teams received data regarding their clinic-specific barriers and facilitators from the needs assessment. Teams also had the option to prioritize which barriers to address using a conjoint analysis approach [7]. Teams who opted to prioritize barriers were given a workspace divided into four quadrants (i.e., low versus high on both dimensions) representing feasibility and importance of addressing clinic-specific barriers. Through collaborative discussion, teams sorted barriers into one of the four quadrants with the intention of first addressing barriers that were deemed of high feasibility and high importance.

Third, although in both conditions objective and clinician self-reported MBC fidelity data was collected from the EHR, this data was consolidated into performance summary reports and disseminated to implementation team members by a research associate 1 day prior to their meetings to guide their work. Data reflected discrete reporting periods between team meetings to allow for an assessment of the team’s impact on MBC. Each team meeting included a discussion of the data report to ensure its accuracy, inform changes to future reports if desired, and guide implementation strategies.

## Limitations

The standardized and tailored methods described in this manuscript include some of the most commonly used implementation strategies such as training, consultation, technology enhancements, audit and feedback, network interventions, and a guideline. However, it is unknown if this specific set of strategies (tailored or standardized) are needed to optimize implementation and sustainment. Also, despite efforts to limit tailoring in the standardized condition, the use of active learning strategies in the training, for example, means that the trainer was likely tailoring content based on barriers identified in the moment versus those identified a priori. Our team is working to compare the differential impact of these methods on clinician and client outcomes in 12 clinics of the United States’ largest behavioral health service provider.

## Additional file

**Additional file 1.** iMBC Consultation form. A standardized case consultation form completed by the clinician prior to the consultation meeting to provide the consultant with context and the specific consultation question.

## Abbreviations

EBP: evidence-based practice
EHR: electronic health record
MBC: measurement based care
PHQ-9: Patient Health Questionnaire
