# Supplementary material for: Implementing measurement based care in community mental health: a description of tailored and standardized methods
Source: BMC Res Notes. 2018 Jan 27;11:76. doi: 10.1186/s13104-018-3193-0 (PMC5787282; doi:10.1186/s13104-018-3193-0)
Supplement: Supplementary file 1 — Additional file 1. iMBC Consultation form. A standardized case consultation form completed by the clinician prior to the consultation meeting to provide the consultant with context and the specific consultation question. [file 13104_2018_3193_MOESM1_ESM.docx]

**Name of Presenter**: **Date:**

**Pseudonym for Client:** **Age**: **Gender**:

**Date of Intake**:

**Number of Sessions:**

**How many times have you administered the PHQ-9 with this client?**

**PHQ-9 Scores (if applicable):**

| **Session** | **Score** |
| --- | --- |
| **Intake** |  |
| **Most Recent** |  |

**Diagnoses (if known):**

**Brief Description of Primary Problem (1-3 sentences):**

**Issue Related to Measurement Based Care (1-3 sentences):**

**How have you attempted to solve this issue?**

**Specific Consultation Question:**
